# Psychological well-being and associated factors among parents of adolescents with non-suicidal self-injury: a cross-sectional study

**DOI:** 10.3389/fpsyt.2023.1253321

**Published:** 2023-09-07

**Authors:** Qian Xia, Yan Zhang, Xuehua Huang

**Affiliations:** Mental Health Center, West China Hospital, Sichuan University, Chengdu, China

**Keywords:** non-suicidal self-injury, parent, anxiety, depression, psychological resilience

## Abstract

**Background:**

Non-suicidal self-injury (NSSI) impacts not only adolescents who engage in it but also their parents. However, there has been limited research into the psychological well-being of these parents. This cross-sectional study aims to investigate the symptoms of anxiety and depression among parents of adolescents who engaged in NSSI in China and the factors associated with them.

**Methods:**

A total of 400 parents of adolescents with NSSI were included. Socio-demographic information of these parents was collected. The Generalized Anxiety Disorder 7-item (GAD-7), the Patient Health Questionnaire 9-item (PHQ-9), and the Connor-Davidson Resilience Scale (CD-RISC) were used to assess symptoms of anxiety, depression, and psychological resilience, respectively.

**Results:**

The majority of the parents were female (83.5%), married (86.3%), and had a senior high school or equivalent and lower education level (67.1%). The study found that 35.3% of the parents experienced clinically significant symptoms of anxiety (GAD-7 ≥ 7) and 40.1% had clinically significant symptoms of depression (PHQ-9 ≥ 7). Parents with larger ages and lower levels of psychological resilience were more likely to experience symptoms of anxiety and depression (*p* < 0.05). Parents who reported bad parent–child relationships showed a higher level of anxiety.

**Conclusion:**

This study provides important insights into the symptoms of anxiety and depression among parents of adolescents with NSSI. Parental age, parent–child relationship, and psychological resilience were associated with symptoms of anxiety and depression in these parents. Implications for the development of interventions aimed at addressing symptoms of anxiety and depression in parents of adolescents with NSSI were discussed.

## 1. Introduction

Non-suicidal self-injury (NSSI) refers to the repetitive and intentional behaviors of damaging one’s own body tissues without an intent to die (1, 2). According to a meta-analysis covering the years 2010–2021, the estimated global lifetime prevalence of NSSI among a non-clinical sample of adolescents is 22.0%, while the estimated global 12-month prevalence rate is 23.2%. Further analysis revealed that the prevalence of repetitive NSSI (20.3%) was notably higher than that of episodic NSSI (8.3%) (3). NSSI can lead to various negative consequences, both physical and psychological. Physically, it can result in tissue damage, scarring, and potential infections (4, 5). Psychologically, individuals who engage in NSSI may experience feelings of guilt, shame, and low self-esteem (6, 7). They may also struggle with emotional regulation and have difficulties in interpersonal relationships (8). Furthermore, NSSI is associated with an increased risk of suicidal ideation and suicide attempts, indicating the seriousness of this behavior (5–7). Therefore, it is crucial to examine the risk factors for the onset of NSSI and to provide appropriate support and interventions to promote the overall well-being of these adolescents.

As primary caregivers, parents of adolescents with NSSI play a critical role in the onset and development of NSSI. Studies have shown that family factors, such as low levels of family psychological functioning and perceived social support, can significantly increase the occurrence of NSSI among adolescents (9). Parental psychological characteristics such as depressive symptoms, distress and expressed emotion have also been identified as potentially contributing to the risk of NSSI (10–12). Moreover, parental behaviors can impact adolescent NSSI, with harsh behavioral control and negative emotional responses increasing the odds of its onset, while parental warmth, such as emotional responsiveness, verbal praise, and support, can reduce the likelihood of its onset (13). Adolescents with a warm parent-adolescent relationship were more likely to have remission in NSSI (14).

Adolescent NSSI not only affects the individual but also has a significant impact on their parents’ psychological well-being. Qualitative studies have revealed that parents experienced negative emotions such as guilt, anger and anxiety, and faced challenges in managing NSSI which can disrupt family functioning and increase financial burden (15–19). If parents’ psychological well-being deteriorates, this may exacerbate conflicts with their adolescents, leading to an increase in NSSI or a lack of quality care for the adolescents. By identifying the contributing factors and addressing them, the psychological well-being of parents, as well as adolescent NSSI, may be improved (20).

To further understand the impact of adolescent NSSI on parents’ psychological well-being, this study investigates the levels of symptoms of anxiety and depression in parents of adolescents with NSSI and examined their associated factors, so that we can better understand the broad impact of NSSI within the family system and take appropriate initiatives to address the NSSI issue.

## 2. Methods

### 2.1. Participants

This cross-sectional study was conducted in a mental health center of a tertiary hospital in Chengdu, China. Inclusion criteria for adolescents were: (1) aged between 10 and 19 years old; (2) having a history of self-injurious behaviors, including but not limited to cutting or burning the skin or banging the head, without an intent to die, within the past 12 months; (3) currently having no psychiatric symptoms such as delusion or hallucination; (4) accompanied by at least one legal guardian. Parents of eligible adolescents were then approached and screened. Inclusion criteria for parents were: (1) the legal guardian of the adolescent; (2) having the ability to understand and speak Mandarin; (3) having no history of diseases that might affect cognitive function, such as head trauma or neurological diseases; (4) being willing to participate in the study. Exclusion criteria for parents were: (1) having a history of mental disorder.

### 2.2. Data collection

All adolescents admitted to the children and adolescents unit of the mental health center from August 2022 to March 2023 were screened for eligibility by three psychiatric nurses. Parents of those eligible adolescents were approached and screened by the same psychiatric nurse. Eligible parents were asked if they were willing to participate in the study. Informed consent was collected from those parents who agreed to participate. All socio-demographic information and scales were collected immediately after obtaining the informed consent.

### 2.3. Measurements

A self-administered questionnaire was used to collect demographic information of parents, including gender, age, marital status, education level, employment status, family economic level, and parent-reported parent–child relationship, with the choices including good, generally fine, and bad. Parental psychological well-being was measured using the Generalized Anxiety Disorder 7-item (GAD-7), the Patient Health Questionnaire 9-item (PHQ-9) and the Connor-Davidson Resilience Scale (CD-RISC), respectively. All scales have been widely used and have good reliability and validity (21–24). Among them, the GAD-7 and PHQ-9 were used to assess symptoms of anxiety and depression, respectively. The higher the score suggests higher level of symptoms of anxiety or depression. The recommended cutoff scores (≥7) for the GAD-7 and PHQ-9 were used to identify whether the parents had clinically significant symptoms of anxiety or depression (21, 22). Parental psychological resilience was assessed using the CD-RISC, with higher scores indicating better resilience.

### 2.4. Statistical analysis

Continuous variables are expressed as mean and standard deviation, whereas categorical variables are expressed as absolute values and percentages. The differences of parental symptoms of anxiety, symptoms of depression and psychological resilience among demographic variables were compared using *t*-test or *χ*^2^ test. The binary logistic regression analysis was performed to examine the influencing factors of parental symptoms of anxiety and depression. All analyses were conducted using Stata v15.0 statistical software and statistical significance was set to a two-sided *p*-value <0.05.

### 2.5. Ethical considerations

The study was ethically approved by the biomedical ethical committee of the hospital where the study was conducted and followed the principles of Helsinki declaration. The aim and content of the study was explained to both adolescents and their parents. No data was collected before informed consent was obtained.

## 3. Results

### 3.1. Parents’ socio-demographic characteristics

In this study, 400 parents participated in this survey, with an average age of 42.4 (SD = 5.4) years. Table 1 provides a descriptive analysis of the demographic characteristics of the parents and their symptoms of anxiety and depression. According to the results, the majority of the parents, who accompanied their children when admitted for NSSI, were female (83.5%), married (86.3%), and had a senior high school or equivalent and lower education level (67.1%). Most parents were employed (55.3%), with moderate family finances (74.5%) and had a good relationship with their children (54.5%).

**Table 1:** Demographic and psychological characteristics of the sample.

Variables	*n*	Percentage
**Demographic characteristics**		
Age groups (years)		
26–39	134	33.5
40–44	123	30.8
45–64	143	35.8
Gender		
Female	334	83.5
Male	66	16.5
Marital status		
Married	345	86.3
Divorced	55	13.8
Education level		
Junior middle school and below	171	42.8
Senior high school or equivalent	97	24.3
Bachelor’ degree or equivalent and above	132	33.0
Employment status		
Unemployed	164	41.0
Working	221	55.3
Retired	15	3.8
Family economic level		
Poor	76	19.0
Moderate	298	74.5
Better	26	6.5
Parent–child relationship		
Good	218	54.5
General	170	42.5
Bad	12	3.0
**Levels of symptoms of anxiety and depression**		
GAD-7		
<7	238	64.7
≥7	130	35.3
PHQ-9		
<7	214	59.9
≥7	143	40.1
Total	400	100.0

### 3.2. Symptoms of parental anxiety and depression and associated factors

Among the 400 parents, 35.3% were identified as having clinically significant symptoms of anxiety, with a mean score of 5.6 (SD = 4.8), while 40.1% were identified as having clinically significant symptoms of depression, with a mean parental symptoms of depression score of 6.0 (SD = 4.7) (Table 1). The mean of parental psychological resilience score was 66.8 (SD = 16.0). Parental age was significantly different between those with and without clinically significant parental symptoms of depression. The average age of parents with clinically significant symptoms of depression was older than that of parents below clinically significant symptoms of depression (*p* = 0.046). A higher percentage of parents with clinically significant symptoms of anxiety had bad parent–child relationships compared to that of parents below clinically significant symptoms of anxiety (*p* = 0.035). Parents with clinically significant symptoms of anxiety or depression had lower psychological resilience scores than those without clinically significant symptoms of anxiety or depression (all *p* < 0.001). These differences are shown in Table 2. According to the results of the binary logistic regression analyses, the present study found that among parents whose children were engaged in NSSI, parental age (*p* < 0.05) was a significant associated factor for both symptoms of anxiety and depression, and the older the parent, the higher the risk of symptoms of anxiety (OR = 1.05) and symptoms of depression (OR = 1.07). The quality of parent–child relationship was another significant associated factor for parental symptoms of anxiety. Parents with bad parent–child relationships were approximately four times more likely to have anxiety (OR = 4.02). Moreover, psychological resilience was also a significant associated factor for parental symptoms of anxiety and depression (all *p* < 0.001), the better the psychological resilience, the lower the risk of symptoms of anxiety (OR = 0.97) and symptoms of depression (OR = 0.97) (Table 3).

**Table 2:** Demographic and psychological resilience differences of parental symptoms of anxiety and depression.

Variables	Symptoms of anxiety				Symptoms of depression			
	Without (*n* = 270)	With (*n* = 130)	*χ*^2^/*t*-test	*p* value	Without (*n* = 257)	With (*n* = 143)	*χ*^2^/*t*-test	*p* value
Age	42.02 (±5.45)	43.15 (±5.37)	−1.948	0.052	41.98 (±5.44)	43.11 (±5.39)	−2.000	0.046
Gender								
Female	221 (81.9)	113 (86.9)	1.638	0.201	215 (83.7)	119(83.2)	0.013	0.909
Male	49 (18.1)	17 (13.1)			42 (16.3)	24(16.8)		
Marital status								
Married	230 (85.2)	115 (88.5)	0.794	0.373	226 (87.9)	119(83.2)	1.727	0.189
Divorced	40 (14.8)	15 (11.5)			31 (12.1)	24(16.8)		
Education level								
Junior middle school and below	117 (43.3)	54 (41.5)	0.758	0.684	111 (43.2)	60(42.0)	1.883	0.390
Senior high school or equivalent	62 (23.0)	35 (26.9)			57 (22.2)	40(28.0)		
Bachelor’ degree or equivalent and above	91 (33.7)	41 (31.5)			89 (34.6)	43(30.1)		
Employment status								
Unemployed	106 (39.3)	58 (44.6)	2.947	0.229	101 (39.3)	63(44.1)	1.996	0.369
Working	156 (57.8)	65 (50.0)			148 (57.6)	73(51.0)		
Retired	8 (3.0)	7 (5.4)			8 (3.1)	7(4.9)		
Family economic level								
Better	21 (7.8)	5 (3.8)	5.510	0.064	46 (17.9)	30(21.0)	3.579	0.167
Moderate	205 (75.9)	93 (71.5)			190 (73.9)	108(75.5)		
Poor	44 (16.3)	32 (24.6)			21 (8.2)	5(3.5)		
Parent–child relationship								
Good	151 (55.9)	67 (51.5)	6.697	0.035	150 (58.4)	68(47.6)	5.973	0.050
General	115 (42.6)	55 (42.3)			102 (39.7)	68(47.6)		
Bad	4 (1.5)	8 (6.2)			5 (1.9)	7(4.9)		
Psychological resilience	69.24 (±15.57)	61.62 (±15.82)	4.560	<0.001	69.66 (±16.19)	61.57 (±14.40)	4.980	<0.001

**Table 3:** Associated factors of parental symptoms of anxiety and depression.

Variable	Symptoms of anxiety			Symptoms of depression		
	OR	95% CI	*p* value	OR	95% CI	*p* value
Age	1.05	(1.00–1.10)	0.033	1.07	(1.02–1.12)	0.006
Gender						
Female	–	–	–	–	–	–
Male	0.68	(0.36–1.30)	0.243	1.02	(0.55–1.87)	0.956
Marital status						
Married	–	–	–	–	–	–
Divorced	0.78	(0.39–1.56)	0.482	1.79	(0.95–3.39)	0.073
Education level						
Junior middle school and below	–	–	–	–	–	–
Senior high school or equivalent	1.74	(0.94–3.20)	0.076	1.70	(0.94–3.06)	0.080
Bachelor’ degree or equivalent and above	1.42	(0.76–2.66)	0.270	1.16	(0.64–2.13)	0.621
Employment status						
Unemployed	–	–	–	–	–	–
Working	0.93	(0.54–1.61)	0.795	0.86	(0.50–1.46)	0.565
Retired	1.33	(0.37–4.73)	0.660	0.76	(0.22–2.70)	0.676
Family economic level						
Better	–	–	–	–	–	–
Moderate	1.75	(0.61–5.02)	0.299	2.56	(0.88–7.43)	0.084
Poor	3.26	(0.99–10.67)	0.051	2.58	(0.79–8.47)	0.118
Parent–child relationship						
Good	–	–	–	–	–	–
Generally fine	0.82	(0.51–1.32)	0.422	1.19	(0.75–1.87)	0.463
Bad	4.02	(1.09–14.83)	0.037	2.39	(0.68–8.40)	0.175
Psychological resilience	0.97	(0.95–0.98)	<0.001	0.97	(0.95–0.98)	<0.001

## 4. Discussion

In recent years, NSSI among adolescents has gained significant attention and has become a growing public health concern worldwide (3, 25). While much of the focus has been on the adolescents themselves, the impact of NSSI on parents is often overlooked. To our knowledge, the present study is the first to investigate the parental symptoms of anxiety and depression of NSSI adolescents in China.

According to the results of the study, a higher percentage of parents with clinically significant symptoms of anxiety (35.3%) and symptoms of depression (40.1%) was identified. These findings are consistent with previous research indicating that parents of adolescents with NSSI often experience high levels of psychological distress and a high risk of symptoms of anxiety and depression. Recent research by Whitlock et al. (26) demonstrated that parents of adolescents with NSSI were more likely to exhibit various forms of objective and subjective strain compared to parents of adolescents without mental health problems. Townsend et al. (20) found that more than half of the parents of adolescents with NSSI were rated as having “mental ill-health”. Therefore, the results of the present study suggest that it is necessary to take care for the mental health of parents of NSSI adolescents and to provide them with psychological interventions.

Our results also found that the parental age, the quality of the parent–child relationship, and psychological resilience appeared to be associated with the prevalence of clinically significant parental symptoms of anxiety and/or depression in this sample of parents. There was a significant difference on parental age between parents with and without clinically significant symptoms of depression. The binary logistic regression analysis showed that the parental age was significant associated factors of clinically significant symptoms of anxiety and depression for parents of NSSI adolescents. Older parents were more likely to suffer from clinically significant symptoms of anxiety and depression. The result suggests that older parents are more susceptible to mental health challenges when dealing with NSSI in their children. This might be attributed to the possibility that as parents age, their children may have been engaging in non-suicidal self-injury (NSSI) for a longer period, thereby extending the duration of the parents’ adverse experiences. Prolonged exposure to adverse experiences has been shown to lead to an increased risk of mental illness, including anxiety and depression (27–29). More studies are needed to verify the specifics of this phenomenon in the future.

In addition, a significant difference in the quality of the parent–child relationship was identified between parents with and without clinically significant symptoms of anxiety. In particular, those parents with clinically significant symptoms of anxiety had a higher percentage of bad parent–child relationships. Previous studies have found that bad parent–child relationships might increase the risk of NSSI in adolescents (13, 30, 31). This finding further suggests that bad parent–child relationships between NSSI adolescents and their parents are associated with an increased risk of parental mental health issues and has a negative impact on parental emotional well-being. Interventions to improve the parent–child relationship may be useful to reduce the risk of adolescents’ NSSI and improve parental symptoms of anxiety and depression.

Furthermore, this study found that among parents of adolescents with NSSI, those with lower levels of psychological resilience had a higher prevalence of clinically significant symptoms of anxiety and depression than those with better levels of psychological resilience. This may be related to the fact that NSSI is a significant negative experience for parents. Parents with higher levels of psychological resilience may be more likely to respond and adjust positively to their children’s NSSI. Previous studies have found that psychological resilience was associated with individuals’ symptoms of depression, symptoms of anxiety, and somatization symptoms when individuals undergo extreme stress or adverse experiences (32–34). People with low psychological resilience usually respond negatively to traumatic stressful events and are also associated with subsequent mental illness (33). Improving psychological resilience may be another effective strategy to address the symptoms of anxiety and depression among parents of adolescents with NSSI.

It is worth noting that our study did not find a significant association between parental gender, employment status, family economic status, and parental symptoms of anxiety and depression. This is somewhat inconsistent with previous studies suggesting that socioeconomic factors may influence mental health among parents of adolescents with mental health problems (35, 36). The discrepancy may be due to the different sample characteristics and cultural contexts, indicating the need for further research in this area. More research is needed to further understand the relationships between socioeconomic factors and mental health among parents of adolescents with mental health problems, and to develop interventions that could support these parents.

There are several limitations in our study. Firstly, the current study employs a cross-sectional design, which means it can only identify associations, not causal relationships. For example, bad parent–child relationship could be a reason for adolescent NSSI or a result. Future longitudinal research is needed to confirm these findings and to explore the potential causal relationships between these risk factors and parental symptoms of anxiety and depression. Secondly, the study did not incorporate the duration and severity of NSSI in adolescents into the analysis, thus it was unable to clarify the direct correlation between NSSI and parental symptoms of anxiety and depression. Thirdly, this study is based on a single-center survey, and thus the findings may not be generalizable. In addition, parents with severe symptoms of anxiety and/or depression were not excluded in this study with the aim to present a whole picture of the psychological well-being of parents of adolescents with NSSI. But these parents were referred to further mental health services for ethical consideration.

In summary, the present study provides important insights into the prevalence and risk factors for symptoms of anxiety and depression among parents of adolescents with NSSI. Parents of adolescents with NSSI were found to have a high prevalence of symptoms of anxiety and depression. Factors such as age, the parent–child relationship, and psychological resilience were identified as significant correlates of parental symptoms of anxiety and depression. These findings have important implications for the development of interventions aimed at addressing symptoms of anxiety and depression in parents of adolescents with NSSI.

## Data availability statement

The raw data supporting the conclusions of this article will be made available by the authors, without undue reservation.

## Ethics statement

The studies involving humans were approved by Ethics Committee of West China Hospital, Sichuan University, Chengdu, Sichuan, China. The studies were conducted in accordance with the local legislation and institutional requirements. The participants provided their written informed consent to participate in this study.

## Author contributions

QX: Conceptualization, Formal analysis, Investigation, Methodology, Writing – original draft, Writing – review & editing. YZ: Conceptualization, Data curation, Investigation, Methodology, Writing – review & editing. XH: Conceptualization, Data curation, Methodology, Writing – original draft, Writing – review & editing.

## Conflict of interest

All authors declare that the research was conducted in the absence of any commercial or financial relationships that could be construed as a potential conflict of interest.

## Publisher’s note

All claims expressed in this article are solely those of the authors and do not necessarily represent those of their affiliated organizations, or those of the publisher, the editors and the reviewers. Any product that may be evaluated in this article, or claim that may be made by its manufacturer, is not guaranteed or endorsed by the publisher.
